# Validation of High-Availability Model for Edge Devices and IIoT

**DOI:** 10.3390/s23104871

**Published:** 2023-05-18

**Authors:** Peter Peniak, Emília Bubeníková, Alžbeta Kanáliková

**Affiliations:** Department of Control and Information Systems, Faculty of Electrical Engineering and Information Technology, University of Zilina, 010 26 Zilina, Slovakia; peter.peniak@feit.uniza.sk (P.P.); emilia.bubenikova@feit.uniza.sk (E.B.)

**Keywords:** Edge, model, Edge device, OPC UA protocol, MQTT protocol, high availability model, IIOT, industry

## Abstract

Competitiveness in industry requires smooth, efficient, and high-quality operation. For some industrial applications or process control and monitoring applications, it is necessary to achieve high availability and reliability because, for example, the failure of availability in industrial production can have serious consequences for the operation and profitability of the company, as well as for the safety of employees and the surrounding environment. At present, many new technologies that use data obtained from various sensors for evaluation or decision-making require the minimization of data processing latency to meet the needs of real-time applications. Cloud/Fog and Edge computing technologies have been proposed to overcome latency issues and to increase computing power. However, industrial applications also require the high availability and reliability of devices and systems. The potential malfunction of Edge devices can cause a failure of applications, and the unavailability of Edge computing results can have a significant impact on manufacturing processes. Therefore, our article deals with the creation and validation of an enhanced Edge device model, which in contrast to the current solutions, is aimed not only at the integration of various sensors within manufacturing solutions, but also brings the required redundancy to enable the high availability of Edge devices. In the model, we use Edge computing, which performs the recording of sensed data from various types of sensors, synchronizes them, and makes them available for decision making by applications in the Cloud. We focus on creating a suitable Edge device model that works with the redundancy, by using either mirroring or duplexing via a secondary Edge device. This enables high Edge device availability and rapid system recovery in the event of a failure of the primary Edge device. The created model of high availability is based on the mirroring and duplexing of the Edge devices, which support two protocols: OPC UA and MQTT. The models were implemented in the Node-Red software, tested, and subsequently validated and compared to confirm the required recovery time and 100% redundancy of the Edge device. In the contrast to the currently available Edge solutions, our proposed extended model based on Edge mirroring is able to address most of the critical cases, where fast recovery is required, and no adjustments are needed for critical applications. The maturity level of Edge high availability can be further extended by applying Edge duplexing for process control.

## 1. Introduction

Industrial applications require the high availability and reliability of devices and systems. Any interruption in production can have serious consequences for the profitability and reputation of the company. Therefore, it is important for industrial applications to possess adequate backup systems and disaster recovery plans for operational processes. The high availability of equipment also means the minimal impact of planned maintenance work on productivity and production time. Finally, high availability also means ensuring minimal risk for employees and their safety when working with industrial applications. Deployment of Industry 4.0 [1,2], in contrast to classical ISA.95 [3,4], with the strictly hierarchical communication of devices via the control systems (PLCs) and information systems, brings completely new approaches and concepts for the overall design, implementation, and high-availability aspects. Smart devices, based on the Industrial Internet of Things (IoT/IIoT) protocols, can be integrated with applications directly and communicate in the peer-to-peer mode independently if used on-premises (local hosts) or via the Internet and Cloud (Software as a Service). However, adequate security, fail-safe features, and the overall orchestration of the communication between applications and IoT platforms should be further enhanced.

**Figure 1:**
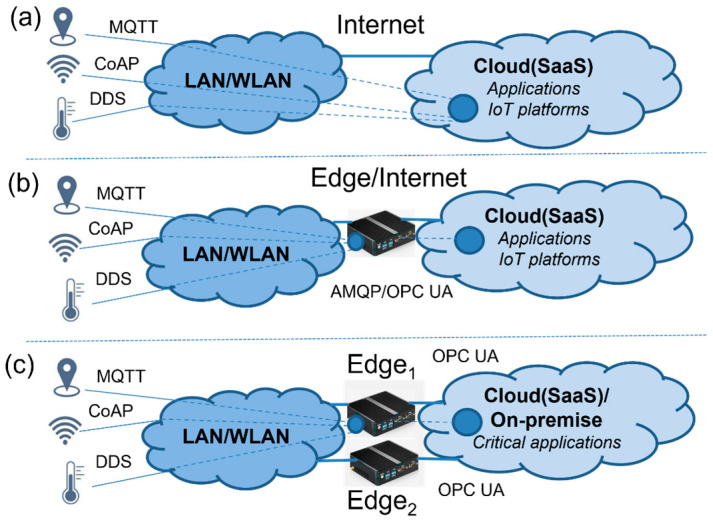
The traditional approach (**a**), Edge-based (**b**), and Edge with High-Availability approach (**c**).

The traditional approach to integrating smart devices with applications—for example, sensors—is shown in Figure 1a. Smart devices can support different application protocols (MQTT, CoAP, DDS, etc.) in order to communicate with applications via TCP/IP connections. The applications could be run either on-premises (local servers) or via a Software as a Service deployment model (SaaS) in the Cloud. The applications perform an evaluation of the delivered data and can create a decision or process the data into information, based on the application logic. In this approach, problems may arise when making decisions in the application, which arise from the need to integrate different protocols, different types of devices, and data synchronization. Another approach can be based on the usage of Edge devices, which is shown in Figure 1b. Edge acts as a generic integration gateway (Middleware), capable of connecting various devices and protocols within the Cloud applications. Such a gateway can be installed as close as possible to the end devices, and is therefore called an Edge device (on the Edge of the Cloud). The Edge device helps to integrate various systems with different application protocols to one connection with the selected protocol and could reduce the overall latency between sensors and applications. It also extends the capability of end devices with the new features, which is known as an Industry 4.0 technique called Digital Twins.

Although the generic Edge devices can address most of the issues, there are still open tasks with respect to industrial communication, the synchronization of sensor inputs, and application requirements for high-availability features. Therefore, our focus will be paid to the creation of the extended Edge model with redundancy, as shown by Figure 1c, with two Edge devices to provide high-availability features, even in the case of Edge device failure. In addition, we will address the integration of IIoT devices with manufacturing applications that expect OPC UA integration. Having created a model, its high availability will be validated on our numerical model and compared with the generic Edge model.

## 2. Related Work

Based on the concept of connecting IOT and IIOT with Cloud services in the literature, we have endeavored to find a link to several reference works.

### 2.1. Cloud Computing

Cloud computing refers to the provision of various services over the Internet, including data storage, servers, databases, networks, and software, as well as its retrieval on demand. A Cloud computing environment provides computing power and storage to offload on-premises systems. However, there are certain disadvantages to using Cloud computing; for example, data transfer to Cloud servers may require significant network bandwidth and may also increase service latency [5]. These disadvantages can be a sensitive issue for some applications. Researchers and companies have proposed two approaches to solve these problems: Edge computing and Fog computing [6]. Cloud computing offers three types of services: infrastructure, platform, and software (IaaS, PaaS, SaaS) [5].

### 2.2. Fog Computing

Fog computing is a decentralized computing infrastructure that stores data, computes data, and contains applications.

It sits somewhere between the data source and the cloud. Similarly, to Edge computing, Fog computing brings the advantages and power of the Cloud closer to where the data is created and acted upon.

### 2.3. Edge Computing

Edge computing is a distributed architecture in which raw data is processed at the edge of the network, as close as possible to the data source, and then the selected data or statistics are transferred to a Cloud server [7,8,9]. There is a growing interest in both academia and industry for the Edge/Fog/Cloud computing of new applications and technologies, such as the IoT, artificial intelligence, machine learning, and process automation. Edge/Fog/Cloud computing represent powerful tools that enable the efficient management and processing of large amounts of data from new technologies and applications.

Edge computing works as an intermediate layer between IoT devices, or IIoT, and the Cloud. The main task of Edge is to mediate the transfer of data from the IoT to the Cloud. It can offer small real-time computing and storage capabilities [9]. The current methods of implementing the Internet of Things (IoT) solutions focus on directly connecting devices to the Cloud, where the data is processed, filtered, or aggregated to increase the business value. However, this approach is insufficient for the manufacturing industry, which requires Edge computing for on-site processing. Industrial devices typically generate significantly more data than standard IoT devices, resulting in delays and increased costs to transfer data to the Cloud. In the industrial context, minimizing response times to critical events and ensuring special security requirements are necessary. Therefore, moving computations to Edge devices in industrial plants can help mitigate these issues and improve the response time and bandwidth efficiency [10]. This means that some data processing and storage processes are moving from the Cloud to Edge. Although the concept of Edge computing is gaining popularity, there is still no consensus on a standardized definition and architecture for Edge computing, [11]. An example of a specific use of Edge in healthcare is the utilization of Edge devices to secure a system that monitors a patient’s health status in a hospital. In the work [12], the issue of data protection using homomorphic encryption is addressed, and Edge is utilized to perform part of the analytical tasks, thereby increasing the performance of encrypted analysis, and reducing the size of the data transmitted to the Cloud. One of the big challenges is deploying Edge in industry. An example of this is the concept that ensures automation in the industry using Edge devices. In [13], the implementation of the Edge Powered Industrial Control concept is realized on an industrial demonstrator using AWS Edge technology. IoT systems can benefit significantly from Edge computing technology, but there are still several challenges related to performance, efficiency, reliability, availability, scalability, security, and privacy [14]. In the following text, we present some interesting solutions that use Edge computing. It is important for Edge computing to remain reliable and fault tolerant when an IoT application is running on a set of Edge networks. It is important to design an efficient and fault-tolerant system for an Edge computing network because of the huge diversity of Edge devices, networks, and computing approaches, as discussed in [15]. In the works, the authors mainly emphasize the speed and accuracy of fault diagnosis, which ensures lower latency and higher availability. Resistance to errors and reliability in Edge computing is also addressed in work [16], where a mobile agent is incorporated, which moves the application to an alternative server in the event of a server failure. Technologies working on software-container-based virtualization have been proposed in [17] for fault tolerance. The work in [18] proposed fault tolerance and backups in Edge cluster networks with support for containers, Kubernetes, and Apache Kafka. Artificial intelligence methods were also applied in Edge implementation and within Edge devices. The mechanism is software-based, supports a software-defined architecture for the IoT, and is robust to various IoT failures and network failures [19]. Some works address the issue of high availability. Document [20] proposes a high-availability architecture in which a Cloud architecture based on templates is designed to automatically configure fault detection and fault recovery methods depending on various service characteristics. In work [21], the authors tackled the minimization of service interruptions and the assurance of the high availability of Edge services by implementing a scheme of real-time internal and external container migration to achieve cooperative processing, load balancing, data backup, and emergency service with switching using Docker technology. The paper [22] proposes a platform where devices in close proximity connect and form a network, called an Edge neighborhood. The platform allows participating devices to utilize the available resources by replicating the metadata from Edge to Edge. Several recent works have proposed the concept of interoperability between Edge/Fog/Cloud in the Internet of Things infrastructures to ensure various Quality of Services (QoS) measures, such as availability and reliability. In works [23,24,25,26,27,28,29], the authors focus on analytical modeling and the evaluation of the availability and reliability of Edge computing using tools such as Markov chains. Work [30] presents a systematic overview of the technologies and methods currently used in federated learning and Edge computing. Works [31,32,33,34] address the integration of blockchain technologies with Edge computing applications.

## 3. Generic Edge Device Model

Let us assume that our Edge device is mainly focused on the integration of various sensors’ data and publishing their results to the Cloud or for processing by on-premises applications, and that there are no specific features required with respect to the Edge computing, fast operation recovery, and high availability. In this case, the Edge device acts as a typical integration gateway.

**Figure 2:**
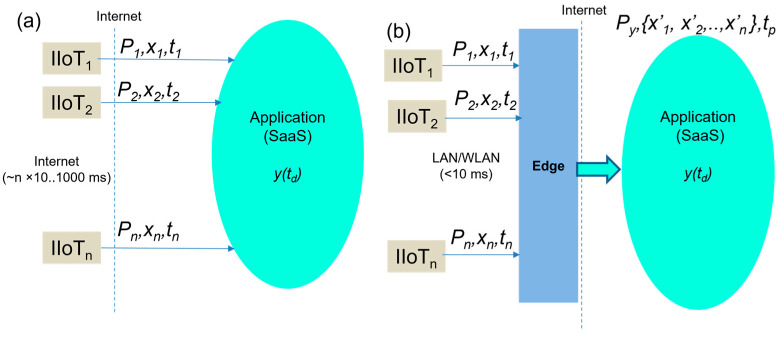
The concept of traditional IIoT integration (**a**) and generic Edge based approach (**b**).

In order to evaluate and compare our extended Edge device models, the generic model has to be created to provide the base for testing, comparison, and to represent generic Edge devices. To create the generic model, let us assume that there is a group of IoT devices: *IIOT* = {*IIOT*_1_, *IIOT*_2_, …, *IIOT_n_*}, where *n* is the total number of distributed IIOT devices. These devices are connected to the Cloud via Internet connection, as shown in Figure 2a. The devices must have assigned the supported application protocol $P=\left\{P_{1},\;P_{2},\ldots ,P_{n}\right\}$, for example, MQTT, CoAP, DDS, etc. Their exchanged data can be formalized as $x=\left\{x_{1},\;x_{2},\ldots ,x_{n}\right\}$ with the assigned timestamp *T*. The timestamp is given by the target application when receiving the data $T=\left\{t_{1},t_{2},\ldots ,t_{n}\right\}$.

The application performs an evaluation of the delivered data and can create a decision or process the data into information based on the application logic y (1). The earliest possible time in which the application is allowed to make a decision *t_d_* is determined by the maximum timestamp (2).

The generic Edge-based approach covers all of the required application protocols and consolidates all of the received values from the sensors, as shown in Figure 2b. It is performed by using the one selected application protocol for Cloud-Edge communication (*P_y_*). The Edge collects all of the sensors’ data $\left(P_{i},t_{i},x_{i}\right)$ within the local network performance conditions (~ms), which is then consolidated to the data that we labeled (*x*′*_i_*) and publishes them according to defined time slots *t_p_* via the Internet. Under the term consolidation, we assume the preparation of all the sensors’ data for publishing via the OPC UA server. The possible publishing time (3) must be calculated as a maximum of the received data timestamps from all sensors (4), which is referred to as the cycle time $t_{c}$ (3) and is extended by any additional over-head time *t_o_* that is needed for data processing by Edge.

(1) $$y:\left[(x_{1},P_{1},t_{1}\right),(x_{2,}P_{2},t_{2}),\ldots ,\;\left(x_{n,}P_{n},t_{n}\right)],$$

(2) $$t_{d}>\max\{t_{1},t_{2},\ldots ,t_{n}\}$$

(3) $$t_{p}=t_{c}+t_{o}$$

(4) $$t_{c}=\max\{t_{1},t_{2},\ldots ,t_{n}\}$$

Therefore, Edge can provide the data from all the connected sensors within the overall publishing time $t_{p}$, as illustrated in Figure 2b. Similar to the traditional approach, the publishing time must be equal or lower than the required decision time (5), (6).

(5) $$t_{p}\leq t_{d},$$

(6) $$y:\left[(x_{1}^{\prime },t_{p}\right),(x_{2}^{\prime },t_{p}),\ldots (x_{n}^{\prime }t_{p}),\;P_{y}].$$

**Figure 3:**
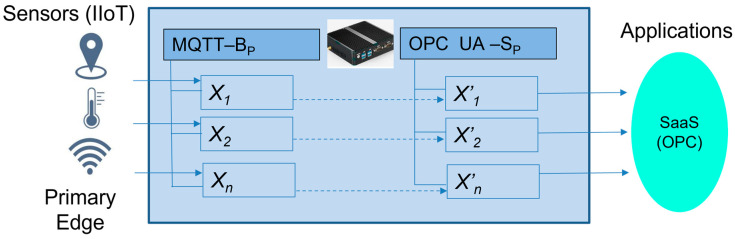
The generic Edge model.

To simplify our basic Edge model, which is shown in Figure 3, let us assume that we only use MQTT as the application protocol for the IIoT devices. The MQTT protocol is typical for telemetry use-cases and sensors with the associated applications. The MQTT broker is an essential part of the basic Edge device model (MQTT-BP) and will be used by sensors or IIoT devices instead of brokers on the application side.

On the contrary, manufacturing applications typically use the OPC UA protocol due to its wide vendors’ acceptance and its advanced cybersecurity features, rich communication capabilities, provided services, and dataspace modeling.

The IIoT values (*x*_1_, *x*_2_, …, *x_n_*) from the MQTT broker items are replicated to the associated variables (*x*’_1_, *x*’_2_, …, *x*’*_n_*) in the OPC UA address space. The OPC UA server (OPC UA-S) then retains all of the values until the next update during the publishing time. Applications have access to the published values via the OPC UA client (“READ” method) from the variables, according to Formula (7):

(7) $$x_{i}\left(t_{i}\right)=x_{i}^{\prime }\left(t_{p}\right).$$

**Figure 4:**
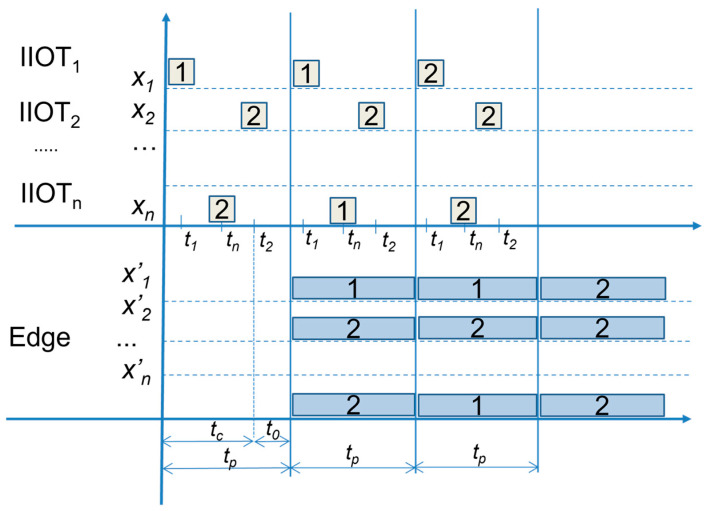
The availability of sensors values *x’_i_* within publishing time *t_p_* by Edge basic model.

MQTT devices can publish and read the data via subscription, but if there is a suddenly broken connection or much higher response time, they can miss published values that are needed for the applications’ evaluation and decision. This can lead to issues with the application logic and the necessity to wait for the next publishing time, which can require additional handling and work-around coding. This can be avoided by keeping the last known value until the next publishing time can be used for the required updates, without interrupting the application logic, which is a native feature of OPC UA server variables and objects (see Figure 4).

## 4. Extended Edge Device Model Device with High Availability

As we presented in our introduction, Edge devices are typically implemented as dedicated devices with a focus on device integration and low latency. In this case, the high-availability features are not solved, and in cases of Edge failure, the device is simply replaced by spare-part Edge. However, manufacturing applications often demand to keep the application running, even in cases of Edge device failure or its unavailability. For this purpose, we had to extend the Edge model to cover the required high-availability features. Let us assume the following possible variants based on the generic Edge device with spare-part and extended Edge device models, which can offer high availability for applications:

- I. Generic Edge model with spare-part device (off-line Edge_2_)
- II. Extended Edge model with mirrored Edge_2_
- III. Extended Edge model with duplexing by Edge_2_.

I. Generic Edge model with spare-part Edge_2_ device

**Figure 5:**
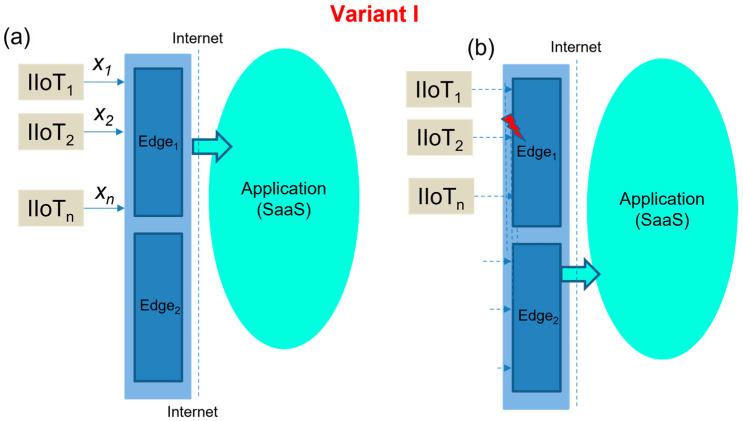
The concept of basic Edge model (**a**) with off-line Edge_2_ (**b**).

This model is virtually identical to the Edge device deployments commonly used at present. Automatic Edge device recovery in cases of failure is not assumed. In the event of an Edge device failure, spare-part Edge will be installed and activated Applications in the Cloud layer are thus exposed to the fact that the Edge device is unavailable and there would be missing sensor data as a consequence. This Edge concept is shown in Figure 5. In Figure 5a we can see basic model and in Figure 5b is spare-part Edge in off-line mode.

**Figure 6:**
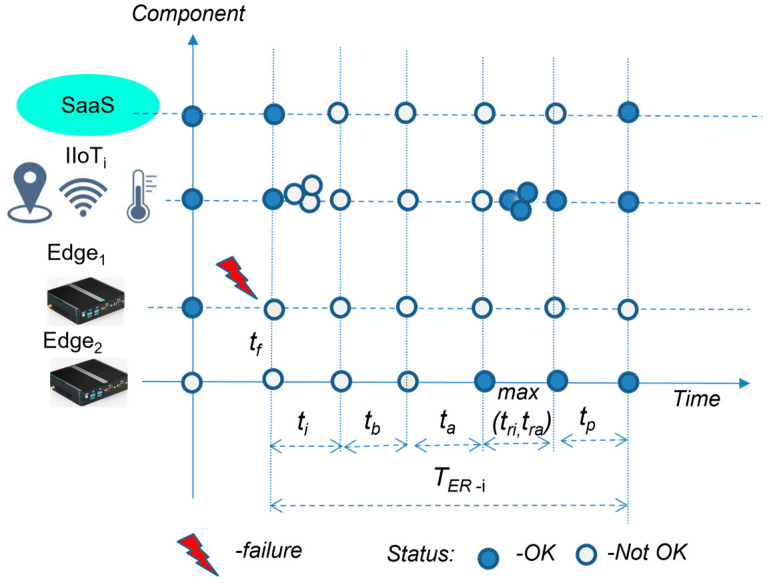
The Edge recovery model.

The Edge recovery time for Variant I (*T_ER-I_*) depends on the activation of the spare-part Edge. This procedure requires HW restart, and the activation of the IP address of the primary Edge, followed by reconnections of all IIoT devices and applications. As soon as Edge_2_ is activated and all devices and applications are reconnected, the application obtains all of the required inputs (sensors’ data) within the next publication time, as expressed by Formula (8). The explanation of the Edge recovery procedure is shown in Figure 6.

(8) $$T_{ER\text{-}I}=t_{i}+t_{b}+t_{a}+max\left\{t_{r1},t_{r2},\ldots ,t_{rn},t_{ra}\right\}+t_{p}$$

where:

*t_i_*—Edge failure identification,
*t_b_*—Edge activation time (HW restart),
*t_a_*—activation of IP address of primary Edge on Edge_2_,
*t_ri_*—reconnection time of *IIoT_i_* device to MQTT broker,
*t_ra_*—reconnection time of application to OPC UA server,
*t_p_*—next publication time of OPC UA server (all sensors’ data collected).

**Figure 7:**
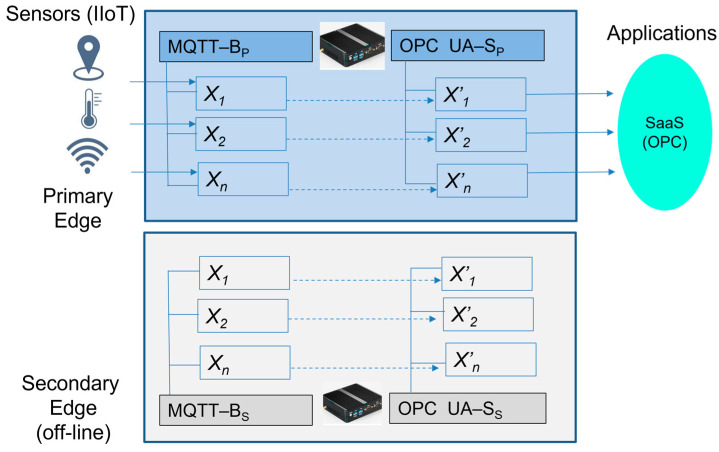
The basic Edge model with off-line Edge_2_.

As explained in Figure 6, during the unavailability of Edge_1,_ all IIoT devices and the applications would lose connection, which is a state that is not suitable (not OK). After the identification of a failure, Edge_2_ is restarted and the IP address of the primary Edge is activated, triggering the reconnection of all IIoT devices and the application itself. As soon as all of the IIoT data are available for publishing, the application can restart the processing of the data. The basic Edge model is shown in Figure 7.

II. Extended Edge model with mirrored Edge_2_ device

This model is based on the two active Edge devices. The primary Edge_1_ device is used by applications and IIoT devices, while the secondary Edge_2_ mirrors all of the sensors’ values from the primary Edge. The secondary Edge is not visible to the IIoT devices and applications, only internally to Edge_1_. Automatic recovery in cases of Edge_1_ failure is implemented and is based on the availability checking of Edge_1_.

**Figure 8:**
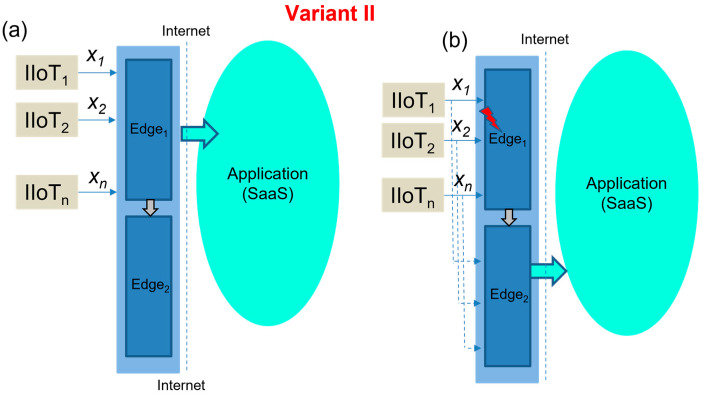
The concept of extended Edge model with mirroring by Edge_2_ in case the right activity (**a**), in case of Edge failure (**b**).

IIoT devices are connected to the primary Edge_1_ (see Figure 8a). The IP address or host name of the primary Edge device is known by all of the connected IIoT devices. Figure 8 illustrates the concept of Edge_1_ mirroring and availability checking by Edge_2_. As mentioned previously, Edge_2_ replicates all of the sensors’ data from Edge_1_. If Edge_1_ fails (see Figure 8b) and is not active on the network, Edge_2_ will identify this event and take over its IP address.

The Edge recovery time for Variant II (*T_ER-II_*) depends on the time required for the identification of an Edge_1_ failure, which is followed by the activation of the IP address on the secondary Edge_2_, while the mirrored sensor data are already available prior to the next publishing time, according to (9):

(9) $$T_{ER\text{-}II}=t_{i}+t_{a}+max\left\{t_{r1},t_{r2},\ldots ,t_{rn},t_{ra}\right\},$$

where:

*t_i_*—identification time of Edge_1_ failure,
*t_a_*—activation of IP address of primary Edge on Edge_2_,
*t_ri_*—reconnection time of *IIoT_i_* device to MQTT broker,
*t_ra_*—reconnection time of application to OPC UA server.

To simplify our model, let us again assume that we only use MQTT as the application protocol for IIoT devices. IIoT devices publish their sensor data (*x*_1_, *x*_2_, …, *x_n_*) to an embedded Edge broker (MQTT-B_p_) via the defined items that are immediately replicated to the associated variables (*x*′_1_, *x*′_2_, …, *x*′*_n_*) of the OPC UA server (OPC fFigureUA-S_p_). The server then keeps all of the values in its object address space until the next update during publishing time. The application (SaaS) has access to the published values via the OPC UA client “READ” method from the variables. The secondary Edge_2_ has the same configuration, but its IP address/hostname is not known to the IIoT devices and applications. Edge_2_ replicates the IIoT values from the primary Edge by subscribing to the same MQTT-B_P_ topics, which are again replicated to the OPC UA server (OPC UA- S_s_) variables (*x*″_1_, *x*″_2_, …, *x*″*_n_)*. Therefore, Edge_2_ is practically able to obtain all the values at the same time as the primary Edge_1_.

(10) $$x_{i}\left(t_{i}\right)=x_{i}^{\prime }\left(t_{p}\right)=x_{i}^{\prime \prime }\left(t_{i}\right).$$

**Figure 9:**
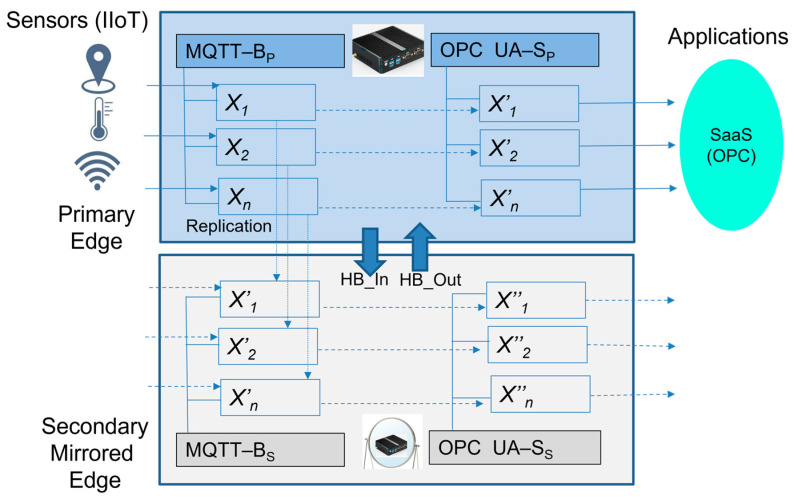
The extended Edge device da model with mirroring.

In cases of primary Edge_1_ failure, the connections of the applications and IIoT devices with Edge_1_ are broken. The secondary Edge_2_ identifies the absence of special heart-beat signals (HB_In) from the primary Edge_1_ as we can see in Figure 9. Edge_2_ takes over the IP address/hostname of the Edge_1_ device. IIoT devices and applications can recover their connection to Edge and have access to MQTT-B_s_ and OPC UA-S_s_ with the replicated values. Formula (11) describes the comparison of *T_ER-I_* and *T_ER-II_* (recovery times) in relation to the $t_{d}\;$(decision time). We can see that the *T_ER-II_* time is minimized using this approach. To maintain the stability of Edge_2_, the heart-beat signal (HB_OUT) prevents Edge_1_ taking the primary IP address in case of its recovery so that the stability and flapping of the connection are mitigated.

(11) $$\;T_{ER\text{-}I}\gg \;T_{ER\text{-}II\;}\leq t_{d}$$

III. Extended Edge model with Edge duplexing

This model applies a pair of Edge devices with independent but identical functions for applications and IIoT devices. In this case, both Edge devices are used equally by all devices and applications, maintaining the overall system redundancy. It means that there are two IP addresses used and two connections established by the applications and IIoT devices.

**Figure 10:**
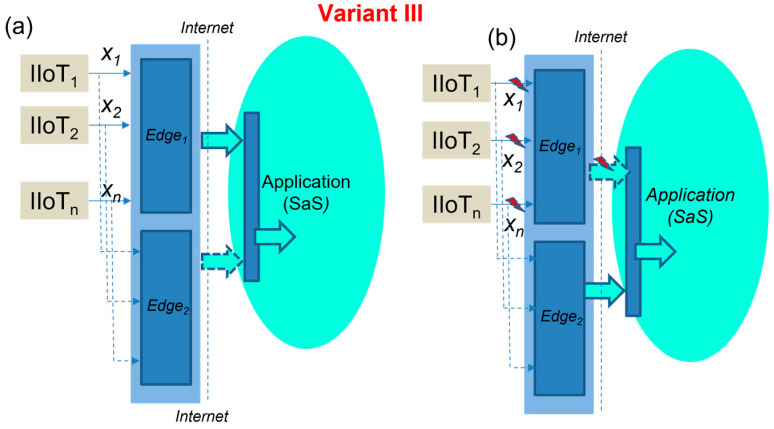
The concept of extended Edge duplexing in case the right activity (**a**), in case of Edge failure (**b**).

**Figure 11:**
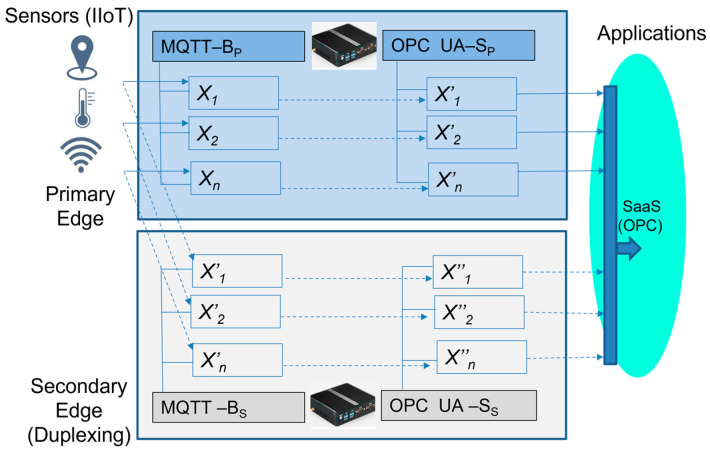
The extended Edge device model with duplexing.

Then, in cases of Edge_1_ or Edge_2_ failure, this solution sustains the operations without any interruption. Figure 10 explains the concept of Edge duplexing in case the right activity (Figure 10a) and in case in failure (Figure 10b) with a defined recovery time, according to (12). The extended Edge model with duplexing is shown in Figure 11.

(12) $$T_{ER\text{-}III}=0.$$

In addition, the received values published by the sensors can have different time stamps (*t_i_*, *t_j_*), based on the network conditions, but are consolidated by both of the Edge device OPC servers in the same publishing time (*t_p_*), as expressed by (13–15).

(13) $$x_{i}\left(t_{i}\right)=x_{i}^{\prime \prime }\left(t_{j}\right)$$

(14) $$x_{i}\left(t_{i}\right)=x_{i}^{\prime }\left(t_{p}\right)$$

(15) $$x_{i}^{\prime \prime }\left(t_{j}\right)=x_{i}^{\prime \prime \prime }\left(t_{p}\right)$$

## 5. The Experimental Workplace for Testing and Validation of Edge Device Models

The proposed models, which are shown in Figure 7 and Figure 9, have been verified in our laboratory. The implementation of the models is based on the Node-RED platform, which is one of the most frequently used products for IoT solutions and can support the creation of various data flows with a broad portfolio of specific nodes, application protocols (TCP, HTTP, MQTT, OPC UA), and supplementary services (such as JSON/XML parsers, file systems, database connectors, etc.).

**Figure 12:**
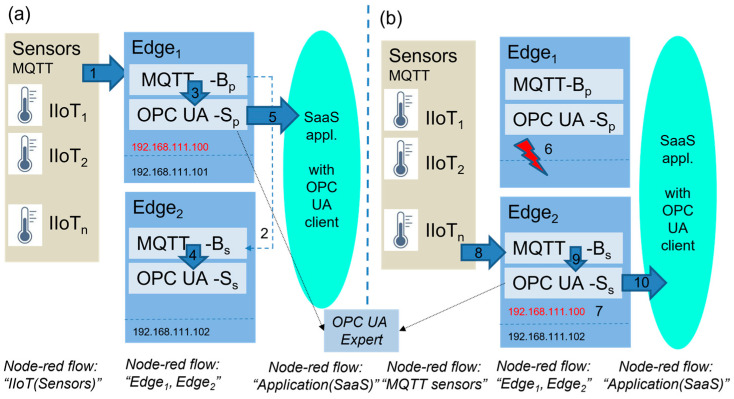
Experimental workplace for validation of high-availability Edge model with default configuration (**a**) and in case of Edge failure (**b**).

The general setup of our simulation experiment is illustrated in Figure 12. All of the sensors are simulated by a Node-RED flow called “IIoT (Sensors)”, which generates an output of sensors at various times and cycles. IIoT_i_ nodes will send the created values to the MQTT client node. The MQTT client publishes the sensors’ values via the assigned topics (*X*_1_, *X*_2_, …, *X_n_*) to the MQTT broker on the Edge device. Applications are represented by another flow, named “Applications (SaaS)”, which simulates the typical application processing of the sensors’ data based on the regular reading of the sensors’ values from the OPC server variables with a defined decision time (*t_d_*). The primary and secondary Edge devices are implemented by dedicated Node-red flows:

- Edge_1_—IP address 192.168.111.101,
- Edge_2_—IP address 192.168.111.102,
- with virtual Edge—IP address 192.168.111.100,

Let us explain the implemented workplace on an example of a test-case for the validation concept of the high-availability Edge device model (Variant 2). We will describe the sequence of steps of the model shown in Figure 12. After restart, Edge devices have a default configuration (see Figure 12a). MQTT sensors publish their values to Edge_1_ via the MQTT broker with the virtual Edge address (1), which is active on the primary Edge_1_. Moreover, Edge_2_ is subscribed to the primary MQTT broker and replicates all of the published items (2). Both Edge devices replicate the MQTT topics (X_1_, X_2_, …, X_n_) to the associated OPC UA server variables (X′_1_, X′_2_, …, X′_n_) (3,4). The application reads the values of the sensors from the OPC UA server items via the virtual Edge device IP address (5). In cases of Edge_1_ failure (6), Edge_2_ takes over the virtual Edge address (7), as shown in Figure 12b. All sensors and OPC UA applications will reconnect or resume communication via the MQTT broker and the OPC UA server of Edge_2_ (8,9,10) based on the virtual Edge address. When Edge_1_ recovers and returns back to the normal mode, it checks if the virtual Edge address is active. If this is a valid case, it does not take over it until restart of the both Edge devices or until the administrator is reset to the default setup.

**Figure 13:**
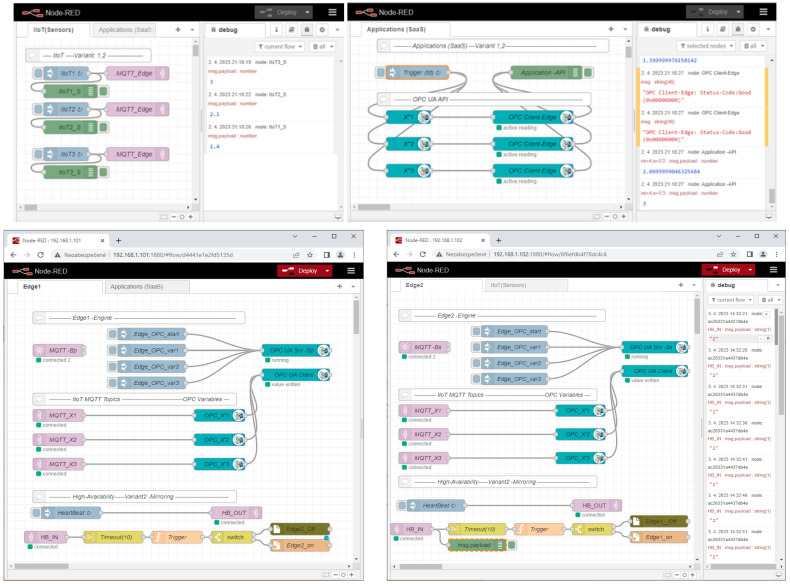
Node-RED flows for experimental validation of high-availability Edge models.

**Figure 14:**
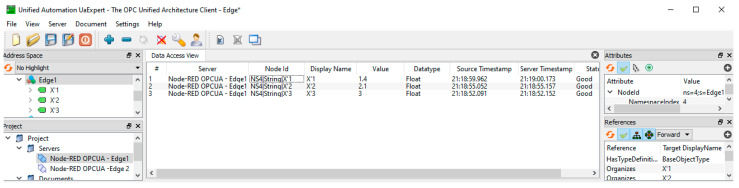
OPC Ua Expert connected to Edge1 OPC UA server with sensor values from Figure 13.

Our experimental workplace with defined Node-RED flows is shown in Figure 13. The number of sensors is limited for the experimental validation to only three devices. All of the sensors’ data are published to the MQTT broker via the defined topics (X_1_, X_2_, X_3_). Edge subscribes to these topics, consolidates them, and writes them into the OPC UA server variables (X_1_, X_2_, X_3_). They can be read by OPC UA clients and the associated applications. OPC UA is capable of providing a timestamp and last valid value for the application, as shown in Figure 14.

The sensors are implemented in Node-red using the standard node “Inject”, named IIoT_1_, IIoT_2_, and IIoT_3_. They regularly inject values, as shown in Figure 13 (debug window). Each sensor generates random values, which are multiplied for tracking, according to (16):

(16) $$x_{i}=i*RND\left(0,1\right).$$

The generation period *T_i_* is configurable and could be set according to the test-cases, for example, according to (17):

(17) $$T_{i}=5*i.\;$$

Edge flows are created with three sections:

1. **Edge engine**—Aedes MQTT Broker for Node-red, OPC UA Server/Client (add-ons) to keep the major services of the Edge system with the option to initialize OPC UA variables.
2. **IoT MQTT Topics-OPC Variables**—Subscribing to the MQTT broker of virtual Edge with writing of received values to the OPC UA server variables.
3. **HighAvailability/Fail-Over-Heart-Beat**—monitoring of Edge_1_ by Edge_2_ based on the heart-beat signal. Takeover of all communication and virtual Edge address in case of its failure.

**Figure 15:**
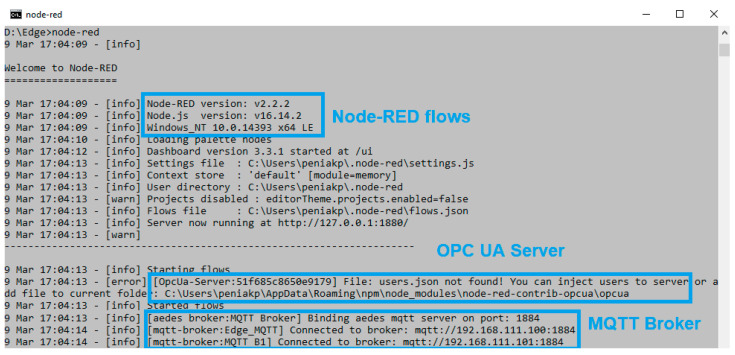
Node-RED console during initialization of Edge flows.

To better explain the Edge engine part, the Node-red console log can be used (see Figure 15). The embedded Node-RED Contrib OPC UA server and MQTT broker from Aedes are activated within the Edge flows during the initialization of Node-RED.

**Figure 16:**
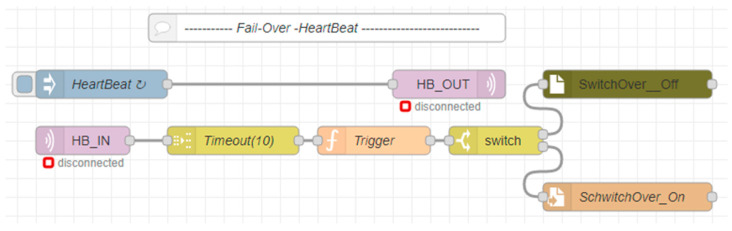
Edge Variant 2 -High-availability/Fail-over with HeartBeat flow.

The second part with IIoT topics and OPC UA variables has already been described; therefore, let us explain the fail-over part of the experimental workplace. If Variant 2 is activated, Edge_2_ subscribes to the HB_IN topic of its internal MQTT broker. The heart-beat signal is regularly published to this topic by Edge_1_ (HB_IN = 2). In cases where the heart-beat value is not published for a longer time than the configurable timeout (10 s), Edge_2_ evaluates this as a failure of Edge_1_ (Edge) and initiates the Trigger function to take over the virtual IP address of Edge_1_ (192.168.1.100), and the Edge status is updated by SwitchOver_On/Edge1_off, as shown in Figure 16.

## 6. The Validation of Edge Device Models in Laboratory

Having implemented an experimental workplace, we were able to test, verify, and validate our Edge device models, with respect to Edge high availability for IIoT devices and applications. In order to evaluate the benefits of the proposed high-availability Edge device models, a comparison with the current models would be necessary. Therefore, our experimental testing and validation will be focused on Variant 1 (Edge basic model) and Variant 2 (Edge extended model with mirroring).

**Figure 17:**
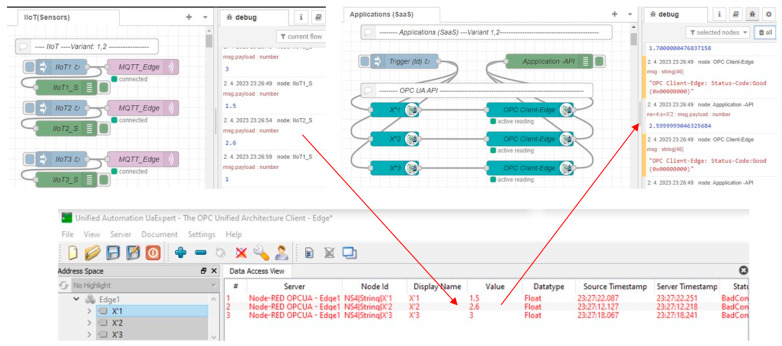
Edge basic model and simulation of Edge1 failure—phase 1: Detection of failure.

Let us focus on the basic Edge model, which does not have embedded high availability. The performed test shows that immediately after Edge_1_ device failure, neither the application nor IIoT devices recognize the failure (MQTT connected, OPC active reading). However, the OPC UA Expert client shows that the quality of the data is bad, with a missing refresh on 23:27 (see Figure 17).

**Figure 18:**
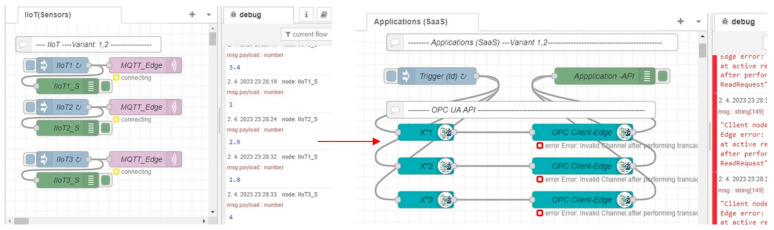
Edge basic model and simulation of Edge1 failure—phase 2: Application malfunction.

**Figure 19:**
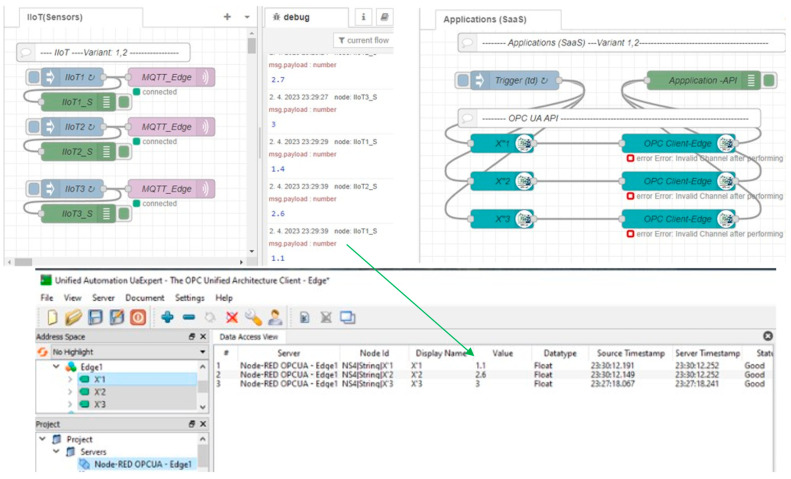
Edge basic model and simulation of Edge1 failure—phase 3: Edge restart.

After the initial phase with the detection of a failure, the application and IIoT devices identified the connection failure with Edge_1_ (MQTT connecting, OPC invalid channel). While the sensors are still generating values, those values are not transferred to the application, so it cannot execute application logic with the sensors’ data after the Edge failure. This phase is shown in Figure 18. To solve this issue, there is a spare-part Edge_2_ kept for Variant 1. Let us assume that Edge_2_ is active (hot-standby mode) and there is only a need for the activation of the Edge_1_ IP address and initialization of the Node-RED software. As soon as Node-RED is reactivated with the IP address of Edge_1_ on the spare Edge_2_, the IIoT devices can reconnect to the MQTT broker and publish their values again, which are replicated by Edge to OPC UA variables; however, the application can still be disconnected until the next reading process is initiated (*t_d_*). This restarting phase with a partially recovered system is shown in Figure 19.

**Figure 20:**
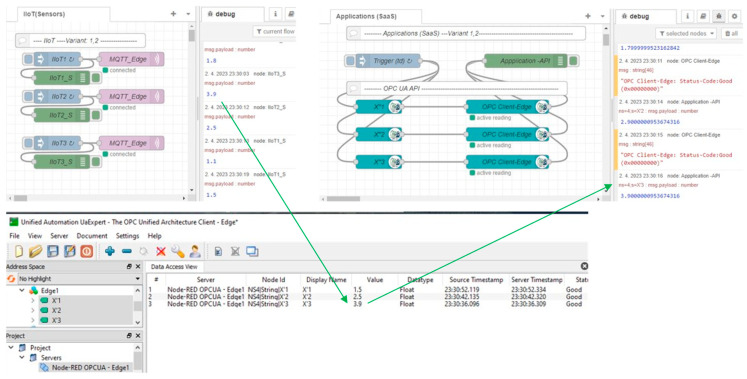
Edge basic model and simulation of Edge1 failure—phase 4: Edge recovery.

Although the spare Edge_2_ helped to recover the incident with the failed primary Edge_1_, there was an overall outage for the application that lasted more than 3 min (See in Figure 20).

**Figure 21:**
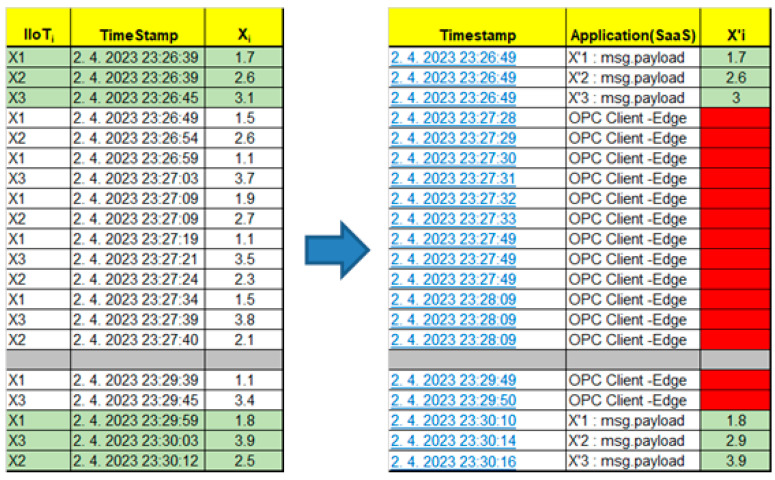
Generated and processed data by application during Edge failure and recovery.

The incident started at 23:27 and ended at 23:30 (we can see in Figure 21) and, so the application did not process the data for more than 3 min. According to Formula (8), we can calculate the time for the Edge recovery:

*T_ER-I_* ~ 180 s

where

*t_i_* = 20 s, *t_a_* = 5 s, *t_b_* + *t_r_* = 145 s, *t_p_* = 10 s.

The consequence of the Edge_1_ failure was that more than 18 calculations and sensor input data were missing during ~3 min of application outage. For a better evaluation, we have introduced the second critical parameter, the number of lost sensor messages *x_L_*:

*x_L_* = 18.

**Figure 22:**
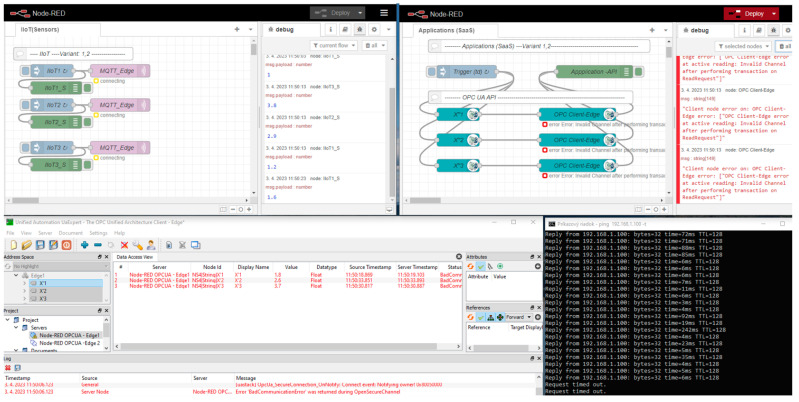
Edge enhanced model—Variant 2: Mirroring—Failure detection.

Apparently, this approach cannot be used for critical applications. Examples of the data generated and processed by the application during the failure and Edge recovery by Variant 1 are shown in Figure 22.

**Figure 23:**
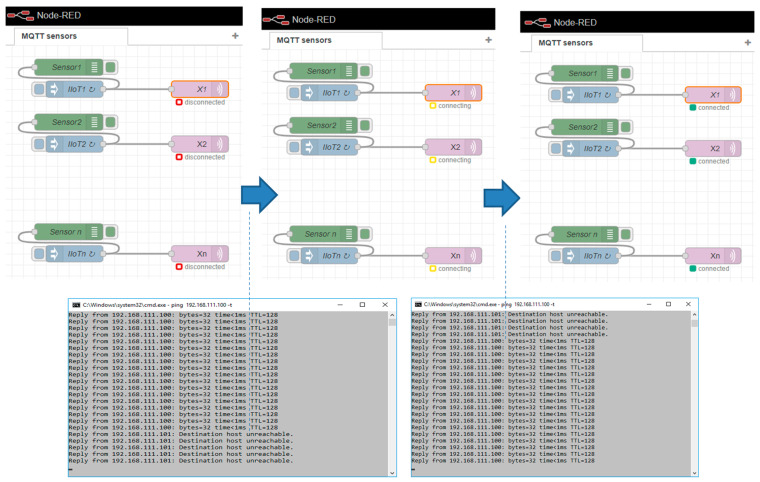
Fail-over process initiated by Edge_2_—Variant 2.

To validate the Edge enhanced model with high availability (Variant 2), we again simulated a failure of the primary Edge_1_. Subsequently, the HB_In signal could not be detected and Edge_2_ activated the virtual IP address by itself. As a result, the virtual IP address was available for the sensors again, which could reconnect, as shown by Figure 23. Finally, the system became stable and the OPC UA application could quickly reconnect.

The high-availability features of the proposed Edge enhanced model (Variant 2) were also confirmed in our experiment. Having simulated a failure of the primary Edge device, the secondary Edge_2_ took over the virtual IP address and the IIoT devices, with the application reconnecting within 9 s. Taking into consideration the configured heart-beat detection time (10 s), the overall time for Edge recovery was ~10× lower than with Variant 1, according to Formula (9):

*T_ER_*_-*II*_ = 19 s,

where

*t_i_* = 10 s, *t_a_* = 5 s, *t_r_* = 4 s.

The OPC UA server maintained the replicated published values from Edge_1_, so there was no loss of data for the application after the OPC UA client was reconnected and the MQTT devices restarted publishing. Therefore, the second critical parameter, which is *x_L_*—number of lost sensor messages, is:

*x_L_* = 0.

**Figure 24:**
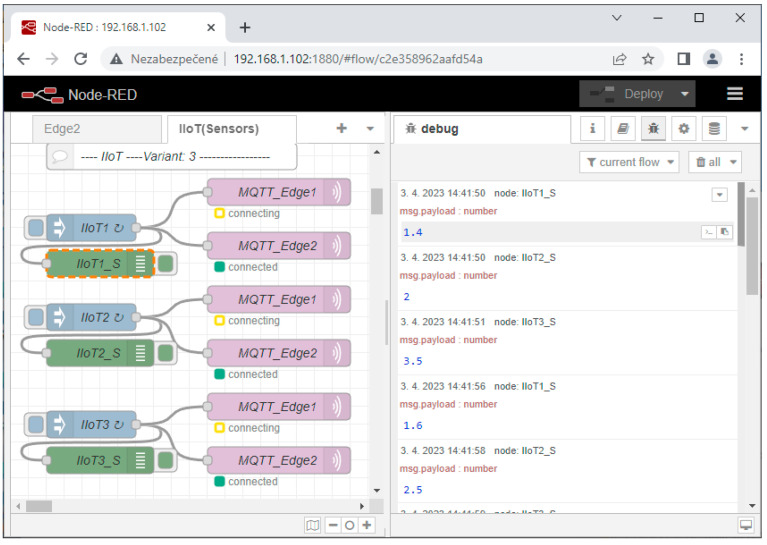
IIoT sensor model for Node-RED—Variant 3 and Edge duplexing.

The enhanced Edge model with Edge duplexing (Variant 3) was not tested or validated. This model requires modified IIoT devices or sensors that are able to maintain parallel MQTT connections to two different MQTT brokers. In addition, the application has to be able to combine two OPC connections. This approach can be applicable for very critical and specific processes within manufacturing, especially if the sensor values can directly influence the process control systems with a high impact on the decisions. Therefore, to prepare future testing and validation models, we have prepared a Node-RED-based model of IIoT devices supporting the Variant 3 requirements, as illustrated by Figure 24.

## 7. Conclusions

Industrial applications require the high availability and reliability of devices and systems. For example, some manufacturing applications require reliable sensor data without any data transmission interruption and subsequent missing data values. The integration of sensors with applications can be realized directly via Internet connection to the Cloud (SaaS) or via an integration Edge device. Our article is focused on the Edge device approach because the potential malfunction of Edge devices can cause the failure of some critical applications, as well as the unavailability of Edge computing results, and can have a significant impact on manufacturing processes.

Our article deals with the design of a suitable Edge model to provide its high availability for applications in an industrial environment with Industrial Internet of Things devices. The high-availability features are not typically considered in generic Edge solutions, which act mainly as an integration gateway, providing device integration with low latency. In cases of Edge failure, there is an expectation that the device is simply replaced by spare-part hardware and activated when possible. However, manufacturing applications have much higher requirements for a fast recovery and reliable operations.

Our main goal is to extend the generic Edge device to support redundancy and fast system recovery in cases of failure. Therefore, we created three variants of Edge models:

Variant I: Generic Edge model without high availability.
Variant II: Extended Edge model with mirroring.
Variant III: Extended Edge model with duplexing.

While Variant I represent the generic Edge model without any focus on high-availability and is used only for comparison with the extended models, the proposed Variants II and III are based on system redundancy with an additional secondary Edge device. Variant II is based on Edge mirroring, which we designed with the aim to minimize the recovery time after Edge device failure through the mirroring of the sensor data on the secondary Edge device. In cases of primary Edge device failure, the secondary one takes over the network configuration and provides the available data (mirrored) to the reconnected applications, while the sensors are also reconnected. Variant III represents the most advanced approach, providing Edge duplexing. In this case, each sensor and application are connected to both Edge devices, providing two channels to receive and process the sensors’ data. With duplexing, any device failure has no impact on critical applications, there is no need to reconnect, and there is no potential loss of the sensors’ data.

For each proposed variant, we have assigned a numerical model with a key performance indicator (KPI) as the recovery time for Edge (*T_ER-x_*) and number of lost IIoT (sensors) messages *x_L_* to evaluate the high-availability maturity level of each variant.

The implementation of the variants was based on the Node-RED platform, which is one of the most commonly used products for IoT solutions and can support the creation of various data flows with a broad portfolio of specific nodes, application protocols, and supplementary services. Our model uses the OPC UA standard, which is the most suitable integration platform for various control systems, manufacturing applications, and IIoT devices. The OPC UA protocol is combined with the more common IoT protocol—the MQTT protocol—to cover an extended product range without embedded OPC support.

Based on our validation and testing, we can state that the generic Edge model (Variant I), which represents the currently available Edge devices, cannot be used for critical manufacturing applications, where IIoT data are needed for decision making processes in semi-real time. The Edge recovery time *T_ER-_*_I_ took several minutes (180 s), with 18 missing messages (*x_L_* = 18), even in the most proactive approach using the active spare Edge (which is not a typical case). With an increased number of sensors or much faster publishing of the sensors’ data, this impact would be even higher.

On the contrary, our proposed extended model based on Edge mirroring (Variant II) was able to address most of the critical cases, where fast recovery is required, and no adjustments are needed for the applications and IIoT devices. The recovery time *T_ER-II_* was roughly 10× lower (19 s) in comparison to the generic Edge model, and the reconnection time *t_ri_* was fast enough not to cause any interruptions or loss of IIoT messages with the sensors’ data (*x_L_* = 0).

In conclusion, we can confirm that our Variant II with Edge mirroring can be applied for a broad range of manufacturing applications, where the reconnection time and fast system recovery is sufficient for application use-cases. There is a clear benefit in the operational aspects of Edge devices, as well in the sensor data availability, in contrast to the Edge generic model and currently available Edge solutions. However, for process control and semi-real time communication, even Variant II does not address all of the challenges. To address this most challenging use-case, there is a need for our Variant III with Edge duplexing. This model works with two Edge devices so that any failure will not be recognized by the IIoT devices or applications. To test and validate this model, we would need to adjust the IIoT devices and applications. This would require further extension and preparation work. In addition, we have limited our validation to only the MQTT protocol. It would be necessary to extend the model for different protocols, such as DDS and CoAP, to obtain validation of the model for all cases. We will need to extend our model to address those requirements in our follow-up articles.

## Data Availability

Not applicable.
